# Consequences of multiple imputation of missing standard deviations and sample sizes in meta‐analysis

**DOI:** 10.1002/ece3.6806

**Published:** 2020-10-07

**Authors:** Stephan Kambach, Helge Bruelheide, Katharina Gerstner, Jessica Gurevitch, Michael Beckmann, Ralf Seppelt

**Affiliations:** ^1^ German Centre for Integrative Biodiversity Research (iDiv) Halle‐Jena‐Leipzig Leipzig Germany; ^2^ Institute of Biology/Geobotany and Botanical Garden Martin Luther University Halle‐Wittenberg Halle Germany; ^3^ Department of Community Ecology UFZ – Helmholtz Centre for Environmental Research Halle Germany; ^4^ Department Computational Landscape Ecology UFZ – Helmholtz Centre for Environmental Research Leipzig Germany; ^5^ Department of Ecology and Evolution Stony Brook University Stony Brook NY USA; ^6^ Institute of Geoscience & Geography Martin Luther University Halle‐Wittenberg Halle Germany

**Keywords:** effect sizes, missing not at random, recommendations, research synthesis, simulated data sets, variance measures

## Abstract

Meta‐analyses often encounter studies with incompletely reported variance measures (e.g., standard deviation values) or sample sizes, both needed to conduct weighted meta‐analyses. Here, we first present a systematic literature survey on the frequency and treatment of missing data in published ecological meta‐analyses showing that the majority of meta‐analyses encountered incompletely reported studies. We then simulated meta‐analysis data sets to investigate the performance of 14 options to treat or impute missing *SDs and/or SSs*. Performance was thereby assessed using results from fully informed weighted analyses on (hypothetically) complete data sets. We show that the omission of incompletely reported studies is not a viable solution. Unweighted and sample size‐based variance approximation can yield unbiased grand means if effect sizes are independent of their corresponding *SD*s and *SSs*. The performance of different imputation methods depends on the structure of the meta‐analysis data set, especially in the case of correlated effect sizes and standard deviations or sample sizes. In a best‐case scenario, which assumes that *SDs and/or SSs* are both missing at random and are unrelated to effect sizes, our simulations show that the imputation of up to 90% of missing data still yields grand means and confidence intervals that are similar to those obtained with fully informed weighted analyses. We conclude that multiple imputation of missing variance measures and sample sizes could help overcome the problem of incompletely reported primary studies, not only in the field of ecological meta‐analyses. Still, caution must be exercised in consideration of potential correlations and pattern of missingness.

## Graphical abstract

Meta‐analyses often encounter studies with incompletely reported variance measures (e.g., standard deviation values) or sample sizes, both needed to conduct weighted meta‐analyses. We present a systematic literature survey on the frequency and treatment of missing data in published ecological meta‐analyses. Simulating the effect of 14 different options to treat missing data in meta‐analysis, we show that multiple imputation of missing variance measures and sample sizes could help overcome the problem of incompletely reported primary studies.

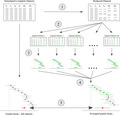

## 1 INTRODUCTION

Research synthesis aims at combining available evidence on a research question to reach unbiased conclusions. In meta‐analyses, individual effect sizes from different studies are summarized in order to obtain a grand mean effect size (hereafter “grand mean”) and its corresponding confidence interval. Most of the analyses carried out in meta‐analysis and meta‐regression depend on inverse‐variance weighting, in which individual effect sizes are weighted by the sampling variance of the effect size metric in order to accommodate differences in their precision and to separate within‐study sampling error from among‐study variation. Unfortunately, meta‐analyses in ecology and many other disciplines commonly encounter missing and incompletely reported data in original publications (Parker, Nakagawa, et al., 2016), especially for variance measures. Despite recent calls toward meta‐analytical thinking and comprehensive reporting (Gerstner et al., 2017; Hillebrand & Gurevitch, 2013; Zuur & Ieno, 2016), ecological meta‐analyses continue to face the issue of unreported variances, especially when older publications are incorporated in the synthesis.

**FIGURE 1:**
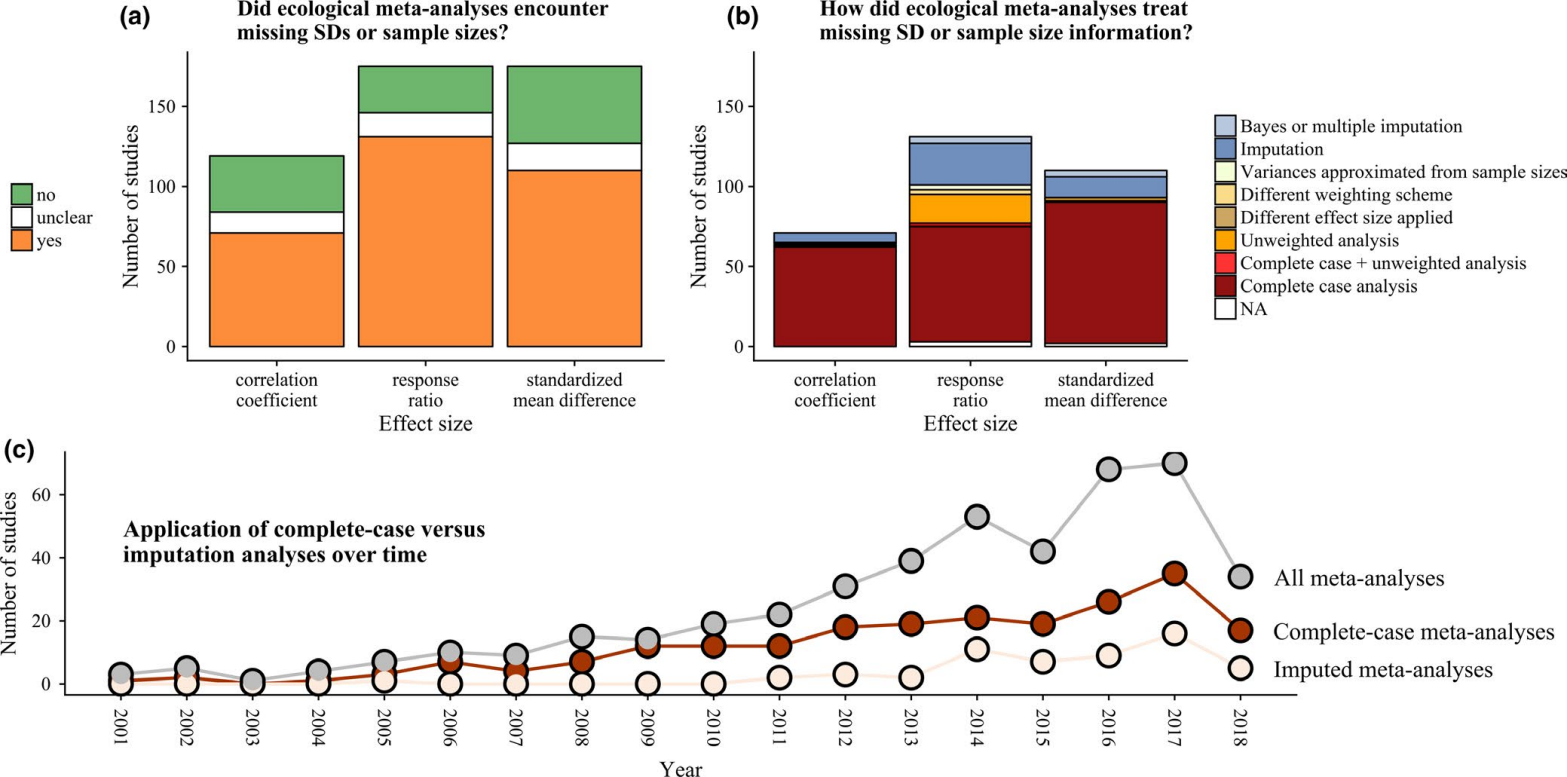
Results of our systematic review on ecological meta‐analyses and their treatment of missing variances and sample sizes in primary studies summarized by 505 ecological meta‐analyses that were published until 23 March 2018 (cf. Data S1 and Appendix S1)

To get an overview about the missing data in meta‐analyses, and to identify how authors of meta‐analysis have dealt with this, we first carried out a systematic survey of the ecological literature. We thereby focused on the most common effect sizes (standardized mean difference, logarithm of the ratio of means, hereafter termed log response ratio, and correlation coefficient (Koricheva & Gurevitch, 2014). Meta‐analysts have essentially four options to deal with missing standard deviations (*SD*s) or sample sizes (*SSs*). The first option is to restrict the meta‐analysis to only those effect sizes that were reported with all the necessary information and thereby exclude all incompletely reported studies. This option (“complete‐cases analysis”) is the most often applied treatment of missing data in published ecological meta‐analyses (see Figure 1). However, at the very least, excluding effect sizes always means losing potentially valuable data. Moreover, if significant findings have a higher chance to be reported completely than nonsignificant results, complete‐case analysis would lead to an estimated grand mean that is biased toward significance (i.e., reporting bias or “file‐drawer problem”(Idris & Robertson, 2009; Møller & Jennions, 2001; Parker, Forstmeier, et al., 2016; Rosenthal, 1979). The second option is to disregard the differences in effect size precision and thereby assign equal weights to all effect sizes. This option (“unweighted analysis”) has also been frequently applied in meta‐analyses of log response ratios (see Figure 1). In the case that no *SD*s are available but *SSs* are reported, a third option is to estimate effect size weights from the SS information alone (see Equation 1, *n_c_* and *n_t_* denominate sample sizes of the control and treatment group, respectively). This “sample‐size‐weighted analysis” depends on the assumption that effects obtained with larger sample size will be more precise than those obtained from a low number of replicates. This weighting scheme has only rarely been applied (see Figure 1).

(1) $${\text{var}}_{\text{approx}}=\frac{n_{t}+n_{c}}{n_{t}\times n_{c}}$$

The fourth option is to estimate, that is, impute, missing values on the basis of the reported ones. In order to incorporate the uncertainty of the estimates, those imputations should be repeated multiple times. When each of the imputed data sets is analyzed separately, the obtained results can then be averaged (“pooled”) to obtain grand mean estimates and confidence intervals that incorporate the heterogeneity in the imputed values.

Various previous studies have suggested that multiple imputations can yield grand mean estimates that are less biased than those obtained from complete‐case analyses (Ellington et al., 2015; Furukawa et al., 2006; Idris et al., 2013; Nakagawa, 2015; Nakagawa & Hauber, 2011). Multiple imputation of missing data can increase the number of synthesized effect sizes and thereby the precision of the grand mean estimate (Idris & Robertson, 2009) or of subgroup mean effect sizes. Imputed data sets permit the testing of hypotheses that could not be tested with the smaller subset of completely reported effect sizes (e.g., on the factors that account for differences in effect sizes).

Despite those advantages, we speculate that the multiple imputation of missing *SD*s and *SSs* has not yet become widely implemented in ecological meta‐analyses, partly because the necessary methods did become available only recently and partly because, from our own experience, it can be difficult to decide on the best imputation method if one assumes that the meta‐analysis data set might harbor hidden correlation structures. Such correlations could comprise relationships between effect sizes and *SD*s or *SSs*. In 1976, Rubin (1976) already defined three distinct processes that could lead to different observed patterns of missing data. If data (in our study *SD*s and *SSs*) are omitted completely by chance, the resulting pattern is coined as *missing completely at random*. If the chance of being omitted correlates with another covariate (in our study with effect sizes), the pattern is called *missing at random*. If the chance of being omitted directly correlates with the value of the data (in our study with SS and *SD* values), this is denoted as *missing not at random*.

Consequently, our second goal was to conduct an evaluation of imputation methods for missing *SD*s or *SSs* studying the most common effect sizes in ecological meta‐analyses (standardizes mean differences, log response ratios, and correlation coefficients (Koricheva & Gurevitch, 2014). Previous studies that compared the effects of different imputation methods focused on a limited number of imputation methods and were conducted on published data sets (Ellington et al., 2015; Furukawa et al., 2006; Idris et al., 2013; Idris & Robertson, 2009; Thiessen Philbrook et al., 2007; Wiebe et al., 2006). In order to systematically determine the effects of correlation structures and patterns of missingness on the performance of different imputation methods, we here simulated data sets that harbored four different correlation structures. This allows to comparing the rigor of the 14 options to treat missing *SD*s and *SSs*, c.f. Table 1. We assessed the performance of those 14 options by comparing the resulting grand means and confidence intervals against the estimates obtained from a fully informed weighted meta‐analysis of the very same data sets. With this approach, we provide the currently most complete overview over the most common and easy to apply options to treat missing values in meta‐analysis data sets. We aim to show how the treatment, proportion and correlation structure of missing *SD*s and *SSs* can drive grand means and their confidence intervals to deviate from the results of fully informed weighted meta‐analyses.

**TABLE 1:** Description of 14 different options to treat missing standard deviations (*SD*s) and/or sample sizes (*SSs*) in meta‐analysis data sets and the conditions under which we expected those options to yield grand means that differ from the results that would be obtained with fully informed weighted meta‐analyses (MCAR—**m**issing **c**ompletely **a**t **r**andom, MAR—**m**issing **a**t **r**andom, MNAR + C – **m**issing **n**ot **a**t **r**andom and *SD*s/SSs correlated to effect sizes)

Option	Description	Assumed conditions that might lead to deviations from fully informed weighted meta‐analyses
(1) Complete‐case meta‐analysis	Omits incompletely reported effect sizes due to which grand mean estimates are expected to exhibit lower precision, that is, larger confidence intervals	Missing values are not MCAR
(2) Unweighted meta‐analysis (Pinheiro et al.,^2018^)	Assigns equal weights to all effect sizes (with reported *SSs*), disregarding the differences in their precision	Effect sizes are related to effect size precision
(3) Sample‐size weighted meta‐analysis	Calculates approximate effect size weights (Equation 1 (Hedges & Olkin, 2014). Not applicable for Hedges' *d*, whose calculation is based on *SSs* (see Appendix S2)	Effect sizes are related to the unaccounted *SD*s in the log response ratio and Hedges' *d*
Imputation of missing values		
(4) Mean value imputation	Fills missing values with the mean of the reported ones and thereby keeps the weights of the completely reported effect sizes	Missing values are outside the range of the reported values and/or not MCAR
(5) Median value imputation	Fills missing values with the median of the reported ones and might be more suitable than mean value imputation if *SD*s or *SSs* follow a skewed distribution	Missing values are outside the range of the reported values and/or not MCAR
Multivariate imputation by chained equations (Azur et al., 2011; Lepkowski et al., 2001; Sterne et al., 2009; White et al., 2011) (with the R‐package used)	The following imputation techniques are applied multiple times to yield separate imputed data sets with separate grand mean estimates which are pooled to obtain meta‐analysis estimates that incorporate the uncertainty in the imputed values (illustrated in Figure 2). Thereby, *SD*s and *SSs* with missing values were treated as dependent variables. *SD*s and *SSs* with complete data as well as mean values and correlation coefficients were treated as predictor variables	
(6) *mice*: Random sample (van Buuren & Groothuis‐Oudshoorn, 2011)	Fills missing values via randomly selecting one of the reported ones	Missing values are outside the range of the reported values and/or not MCAR
(7) *mice*: Linear regression (van Buuren & Groothuis‐Oudshoorn, 2011)	Fills missing values with predictions that are obtained from linear models	Missing values are MNAR
(8) *mice*: Predictive mean matching (van Buuren & Groothuis‐Oudshoorn, 2011)	Estimates linear models and fills missing values with those reported values that are closest to the predictions. Imputed values are thereby restricted to a subset of the reported ones	Missing values are outside the range of the reported values and/or MNAR
(9) *mice*: Classification and regression trees (van Buuren & Groothuis‐Oudshoorn, 2011)	Implements a machine‐learning algorithm that seeks cutting points in the set of supplied predictor variables in order to divide the meta‐analysis data set into homogenous subsamples. Fills missing values with random samples from the reported values that are assigned to the same subgroup as the predictions ones. Like predictive mean matching, imputed values are thereby restricted to a subset of the reported ones	Missing values are outside the range of the reported values and/or MNAR
(10) *mice*: Random forest (van Buuren & Groothuis‐Oudshoorn, 2011)	Implements a random forest algorithm (Breiman, 2001) and fills missing values with average predictions from 10 classification and regression trees that are based on 10 random subsets of the predictor variables. This method shares many features with the classification and regression tree imputation but the imputed values exhibit a larger variability	Missing values are outside the range of the reported values and/or MNAR
(11) *mi*: Bayes predictive mean matching (Su et al., 2011)	Fits Bayesian generalized linear models to fill missing values with those reported values that are closest to the predicted ones. Like predictive mean matching, imputed values are thereby restricted to a subset of the reported ones	Missing values are outside the range of the reported values and/or MNAR
(12) *Amelia*: Bootstrap expectation maximization (Honaker et al., 2011)	Draws multiple bootstrap samples from the supplied data and calculates separate posterior maxima. The distribution of these maxima is then used to fill the missing values. In order to yield reliable imputations, this algorithm assumes multivariate normality and MCAR or MAR	Missing values are MNAR
(13) *missForest*: Nonparametric random forest (Stekhoven & Bühlmann, 2012)	Iterates the random forest algorithm (Breiman, 2001) until a certain convergence criterion is fulfilled	Missing values are outside the range of the reported values and/or MNAR
(14) *Hmisc*: Additive regression plus bootstrap predictive mean matching (Frank, & Harrell, 2018)	Draws multiple bootstrap samples from the supplied data and fits separate additive regression models to obtain averaged predictions for the missing values. These missing values are then filled with those observed values that are closest to the predicted ones. Like predictive mean matching, imputed values are thereby restricted to a subset of the reported ones	Missing values are outside the range of the reported values and/or MNAR

## 2 MATERIALS AND METHODS

### 2.1 Systematic literature survey

On 12 March 2018, we executed search queries in the Web of Science and google scholar with the search term (meta‐analys* OR meta‐regression*) AND ecolog*. Google scholar results were compiled with the software *Publish or Perish 6* (Tarma Software Research Ltd, 2007). The 2,626 publications we identified were screened for the following inclusion criteria: (a) the research field was ecology (excluding medical, social, financial, and ecosystem service studies), (b) the authors conducted an original meta‐analysis that was based on summary statistics from previous publications (excluding theoretical, methodological, commentary, raw data analysis, and update studies), (c) the study quantified effect sizes as either response ratios, mean differences or correlation coefficients and (d) the authors could or should have applied a weighting scheme to summarize those effect sizes.

The 505 studies that met these criteria were then screened in order to extract: (a) the year of their publication, (b) the effect size applied (response ratio, mean difference, or correlation coefficient), (c) whether or not the authors encountered primary studies with missing variance or sample size information, and (d) how the authors dealt with this missing information. Cases where the authors were vague with stating how they dealt with missing data (e.g., statements such as “we extracted all available data”) were classified as *missing data encountered*. The Literature search, inclusion criteria, data extracted, and the Preferred Reporting Items for Systematic Reviews and Meta‐Analyses (PRISMA) are reported in the Appendix S1

### 2.2 Simulation of missing *SDs* and/or *SSs* in meta‐analysis data sets

We assessed the effects of 14 options to treat increasing proportions of missing *SDs and/or SSs* on the grand mean and the corresponding confidence interval.

#### 2.2.1 Data‐generating mechanism

We created two types of meta‐analysis data sets. The first data set was created to calculate effect sizes that summarize mean differences between control and treatment groups. The second data set was created to analyze effect sizes that summarize mean correlation coefficients. Each data set consisted of 100 rows representing 100 hypothetical studies with separate means, *SD*s and *SSs* for the control and treatment group (for the mean difference data sets) and separated correlation coefficients and *SSs* (for the correlation coefficient data sets). To reduce random noise and obtain more stable results, we created ten separate mean difference data sets and ten separate correlation coefficient data sets. Mean difference data sets were created with the following data‐generating mechanisms. Mean values for the control groups were randomly drawn from a truncated normal distribution with mean = 1, *SD* = 0.25, and lower limit = 0.001. Mean values for the treatment groups were randomly drawn from a truncated normal distribution with mean = 2, *SD* = 0.5, and lower limit = 0.001. *SD* values for the control groups were randomly drawn from a truncated normal distribution with mean = 0.25, *SD* = 0.125, lower limit = 0.01, and upper limit = 1. *SD* values for the treatment groups were randomly drawn from a truncated normal distribution with mean = 0.5, *SD* = 0.25, lower limit = 0.01, and upper limit = 1. SS values for the control and the treatment groups were both drawn from a truncated Poisson distribution with *λ* = 10 and lower limit = 5. Correlation coefficient data sets were created with the following data‐generating mechanisms. Correlation coefficient values were drawn from a truncated normal distribution with mean = 0.5, *SD* = 0.125, lower limit = −1, and upper limit = 1. SS values were drawn from a truncated Poisson distribution with *λ* = 10 and lower limit = 5.

In all data sets, we simulated missing data by either randomly or nonrandomly deleting between 10% and 90% of the *SD*s, *SSs* or both in the mean difference data sets and between 10% and 90% of the *SSs* in the correlation coefficient data sets (in steps of 5%). Within each data set row, we thereby deleted the *SD*s in both, the control and treatment group and we independently deleted the *SSs* in both, the control and treatment group. With these deletions, we constructed the following four deletion/correlation scenarios, visualized in Appendix S2:

- *SD*s and/or *SSs* were deleted completely at random (**MCAR**, **m**issing **c**ompletely **a**t **r**andom), and there were no correlations in the data sets.
- The chance of deleting *SD*s and/or *SSs* increased with decreasing effect size values (**MAR**, **m**issing **a**t **r**andom). All effect sizes were ranked in decreasing order and the chance of deletion linearly increased with the rank position of the effect sizes. No further correlations were introduced.
- The chance of deleting *SD*s and/or *SSs* increased with increasing *SD*s and decreasing SSs (**MNAR**, **m**issing **n**ot **a**t **r**andom). We ranked the summed *SD*s (*sd_t_* + *sd_c_*) in increasing order (corresponding to a lower precision) and ranked the summed *SSs* (*n_t_* + *n_c_*) in decreasing order (corresponding to a lower sample size). The chance of deletion linearly increased with the rank position of the summed *SD* and SS values. Effect sizes with a lower precision or sample size thereby had a higher change of their *SD*s and *SSs* being deleted. No further correlations were introduced.
- Effect size values were paired with effect size precision (i.e., sorted so that larger effect sizes had smaller *SD*s and larger *SSs*). *SD*s and/or *SSs* were **m**issing **c**ompletely **a**t **r**andom (**corMCAR**). This hypothetical scenario might happen in meta‐analyses across different study designs that impact both the obtained effect size and its precision (e.g., due to the different possibilities to account for additional drivers of effect sizes in experimental versus observational studies).

In total, we created 2,560 data sets: four deletion/correlation scenarios, four types of deleted data (*SD*s, *SSs*, or both for mean difference data sets and only SSs for correlation coefficient data sets), 10 randomly generated data sets and 16 deletion steps (10%–90% of values deleted).

#### 2.2.2 Handling of missing data

**FIGURE 2:**
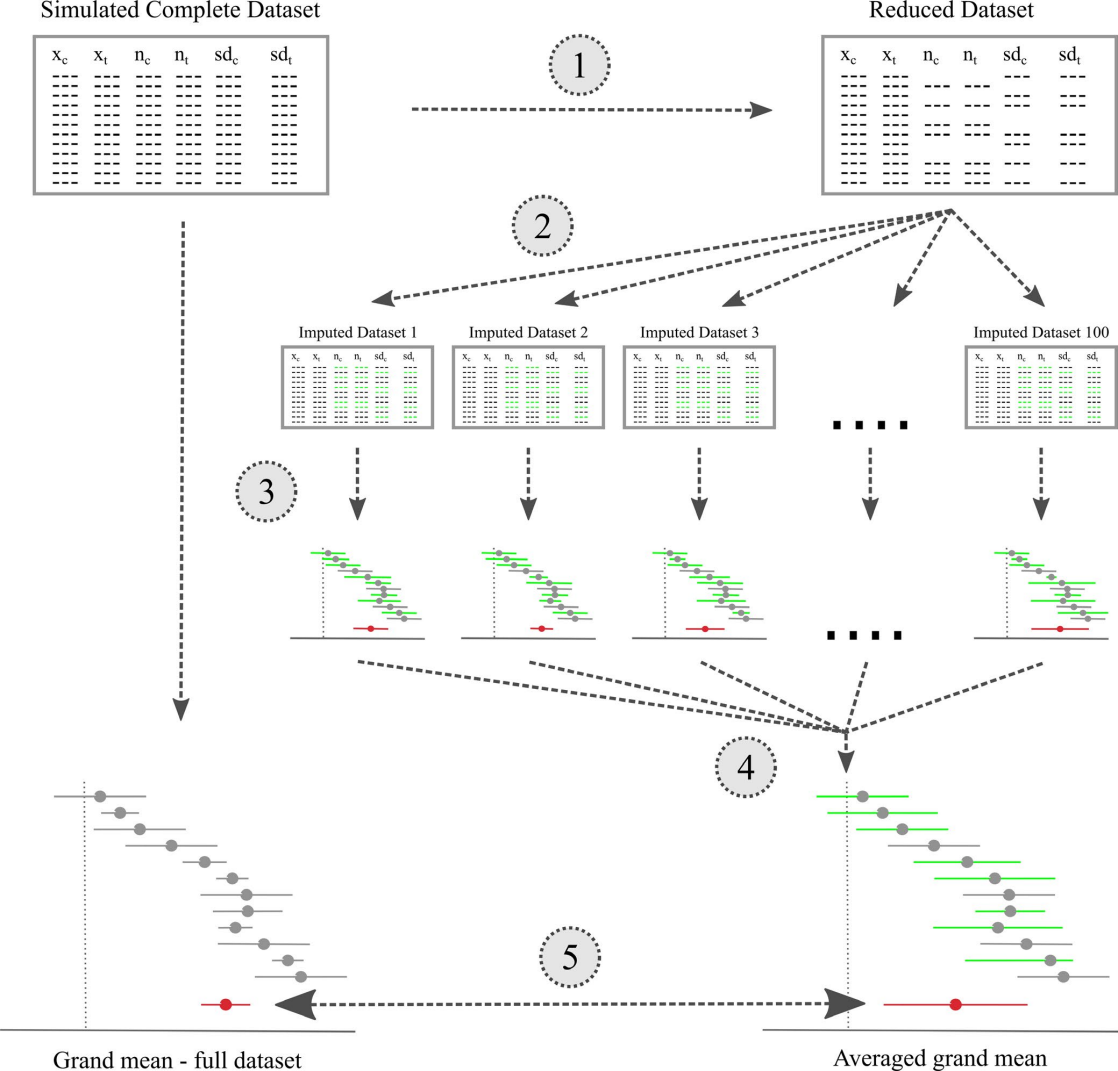
Workflow to compare the effects of the multiple imputation of deleted standard deviations (*SD*s) and sample sizes (*SSs*) with a meta‐analysis of a complete data set. (1) We deleted between 10% and 90% of the *SD*s, SSs or both in the control and treatment groups of an artificial data set. (2) The deleted values were imputed (in green) via multiple imputations (100 times), all done with the same imputation method. (3) Each of the 100 data sets was analyzed with a separate linear mixed‐effects meta‐analyses (imputed values in green). (4) The resulting 100 grand means, and confidence intervals were averaged according to Rubin's rules (Rubin 1987) in order to obtain single estimates. (5) These estimates were compared with the results of an analysis of the complete data set (i.e., without missing values)

To each of the 2,560 data sets, we separately applied one of the outlined 14 options to handle missing *SD*s, and/or *SSs* in meta‐analysis data sets (Table 1). For the sample‐size weighted meta‐analysis, we assigned approximate variance measures to each effect size, according to Equation 1. Our general workflow to fill missing values via multiple imputations is illustrated in Figure 2. We generally restricted imputed *SD*s to range between 0.01 and 1 and imputed *SSs* to be ≥5. Those restrictions were applied to prevent implausible (e.g., negative) imputations and guarantee convergence of subsequent linear mixed‐effects models. Data were imputed in the following order: *SD*s of the treatment group, *SD*s of the control group, SS of the treatment group, and *SSs* of the control group. Changing this imputation sequence had virtually no effect on the results. For the bootstrap expectation maximization imputation, we only imputed data sets with up to 60% of missing values because the algorithm frequently crashed above this threshold. Similar to White et al. (2011) and Ellington et al. (2015), we repeated all imputation methods 100 times (thus “multiple imputations”) to obtain 100 imputed data sets.

#### 2.2.3 Effect sizes

After applying the outlined 14 options to handle missing *SD*s and/or *SSs* (Table 1), we calculated the three most prominent effect size measures in ecological meta‐analyses together with their respective variance estimates where possible/necessary. With the mean difference data sets, we calculated the small‐sample bias‐corrected log response ratio (Lajeunesse, 2015) (hereafter log response ratio) and Hedges' *d*. With the correlation coefficient data sets, we calculated Fisher's *z* (see Appendix S2, for the equations applied).

#### 2.2.4 Grand mean estimates

For every data set (including complete, unweighted, approximately weighted, and imputed data sets), we calculated the grand mean effect size and its corresponding approximated 95% confidence interval with a linear intercept‐only mixed‐effects model. Thereby, the effect size from each data set row was treated with a random effect and weighted by the inverse of its corresponding or approximated variance estimate (*rma* function in the *metafor* package (Viechtbauer, 2010). For every imputation method and every percentage of missing *SD*s and/or *SSs*, the resulting 100 grand mean and 95% confidence interval estimates were averaged under consideration of the uncertainty that arose from the multiple imputations (using Rubin's Rules (Rubin, 1987) as implemented in the *mi.meld* function of the *Amelia* package (Honaker et al., 2011).

#### 2.2.5 Performance measures

We evaluated the effects of the different options to handle missing *SD*s and/SSs in terms of the obtained grand mean and the width of the corresponding 95% confidence interval against reference values obtained with a weighted meta‐analysis on the complete data sets (hereafter fully informed weighted meta‐analysis). Deviation in the grand mean was quantified as the obtained grand mean estimate minus the estimate from the fully informed weighted analysis. Deviation in the confidence interval was quantified as the obtained width of the confidence interval minus the width from a fully informed weighted analysis. We then graphically summarized the trends in the grand mean and confidence interval from using different options to handle increasing proportions of missing *SD*s and/or *SSs*. We refrained from using performance measures, such as the root‐mean‐square error, to compare the different options to handle missing data because we aimed at demonstrating general and nonlinear trends. Since some of the imputation models failed to converge above a threshold of ca. 60% of missing data this would render performance measures infeasible above this threshold.

All analyses were conducted in *R* (R Core Team, 2018) using *ggplot2* for graphical representations (Wickham, 2009). The *R*‐scripts used to simulate the data sets, delete and impute missing *SD*s and/or *SSs* are available at github.com/StephanKambach/SimulateMissingDataInMeta‐Analyses. Script number three can be used to quickly compare the effects of the 14 options to treat missing *SD*s and/or *SSs* on the grand mean of any supplied meta‐analysis data set that should be summarized with the log response ratio, Hedges' *d* or Fisher's *z*.

## 3 RESULTS

### 3.1 Systematic literature survey

In the compiled data set of 505 published ecological meta‐analyses, 35% used log response ratios, 36% used standardized mean differences, 24% used correlation coefficients, and 5% used a combination of the three investigated effect size measures. At least 64% of the investigated ecological meta‐analyses encountered missing variance measures or sample sizes in the primary literature (Figure 1). Most often, the exact number of incompletely reported primary studies was not stated. Forty‐five percent of meta‐analyses just noted that they included only completely reported primary studies. The highest percentage of missing data was reported for those studies that summarized response ratios. For 10% of the studies, we could not determine whether they were affected by missing data. Most studies simply omitted incompletely reported studies from their analyses (complete‐case analysis). A minor fraction of imputed missing data and only two percent of the reviewed meta‐analyses (9 out of 505) applied multiple imputations or Bayesian models to account for imputation uncertainty. The proportion of meta‐analyses that omitted incompletely reported studies versus those that imputed missing data did not change with the publication year (Figure 1).

### 3.2 Visualization of the simulation results

**FIGURE 3:**
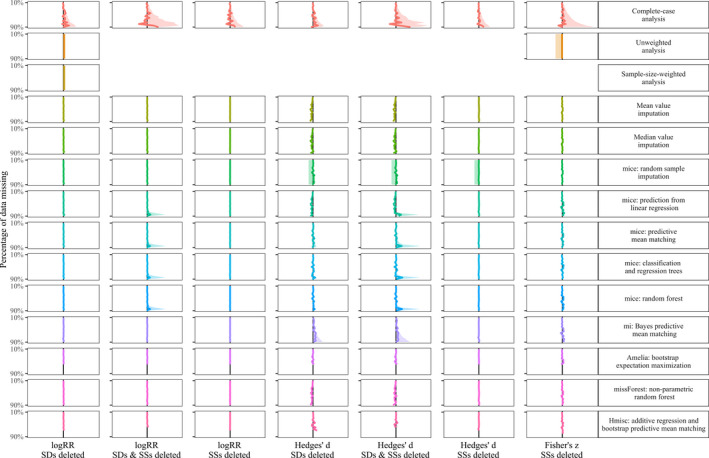
Effects of imputing *SD*s and SSs that are missing completely at random (MCAR) on the grand mean (colored line) and confidence interval (shaded area) with respect to the results of fully informed weighted meta‐analyses. Rows show results for the 14 methods to treat missing values (c.f. Table 1). Columns show result for the log response ratio, Hedges' *d* and Fisher's *z* effect sizes with 10% (top) up to 90% (bottom) of standard deviations (*SD*s) and/or sample sizes (SSs) removed. Each panel shows the deviation of the grand mean and its approximated 95% confidence interval (divided by two for better visibility) from the results obtained with a fully informed weighted meta‐analysis. Deviations to the right indicate lower values and deviations to the right indicate higher values

**FIGURE 4:**
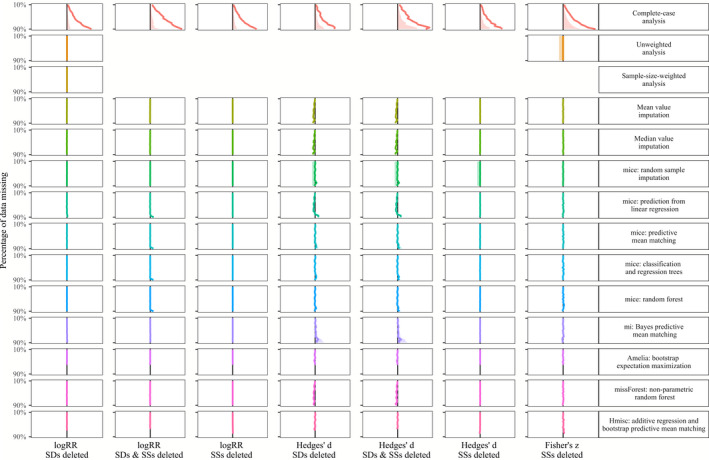
Effects of imputing *SD*s and SSs that are missing at random (MAR) on the grand mean (colored line) and confidence interval (shaded area) with respect to the results of fully informed weighted meta‐analyses. Rows show results for the 14 methods to treat missing values (c.f. Table 1). Columns show result for the log response ratio, Hedges' *d* and Fisher's *z* effect sizes with 10% (top) up to 90% (bottom) of standard deviations (*SD*s) and/or sample sizes (SSs) removed. Each panel shows the deviation of the grand mean and its approximated 95% confidence interval (divided by two for better visibility) from the results obtained with a fully informed weighted meta‐analysis. Deviations to the right indicate lower values and deviations to the right indicate higher values

**FIGURE 5:**
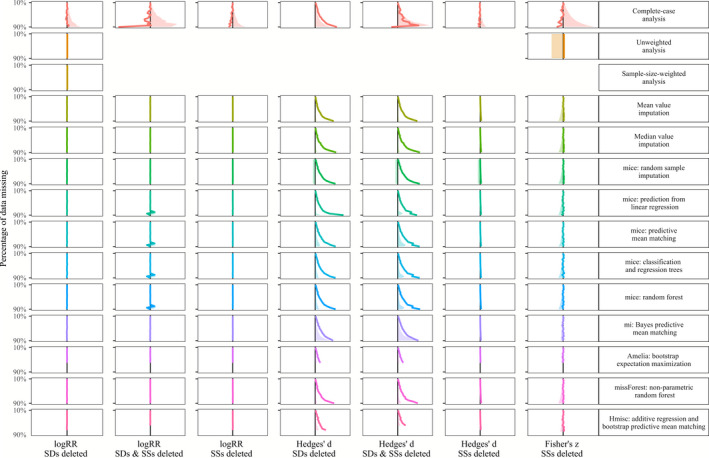
Effects of imputing *SD*s and SSs that are missing not at random (MNAR) on the grand mean (colored line) and confidence interval (shaded area) with respect to the results of fully informed weighted meta‐analyses. Rows show results for the 14 methods to treat missing values (c.f. Table 1). Columns show result for the log response ratio, Hedges' *d* and Fisher's *z* effect sizes with 10% (top) up to 90% (bottom) of standard deviations (*SD*s) and/or sample sizes (SSs) removed. Each panel shows the deviation of the grand mean and its approximated 95% confidence interval (divided by two for better visibility) from the results obtained with a fully informed weighted meta‐analysis. Deviations to the right indicate lower values and deviations to the right indicate higher values

**FIGURE 6:**
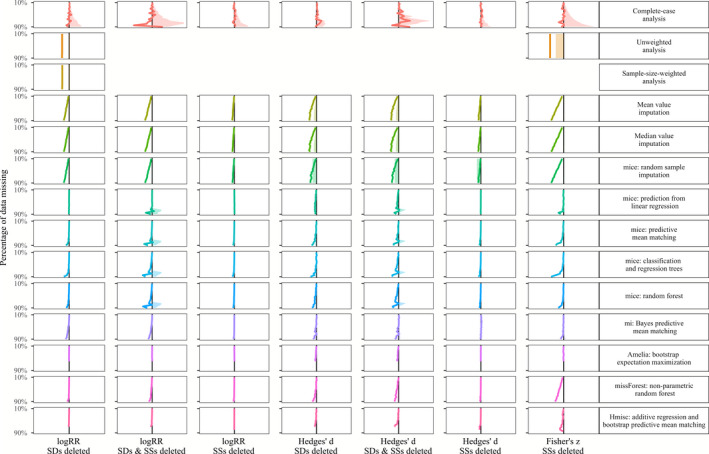
Effects of imputing *SD*s and SSs that are correlated with effect sizes and missing completely at random (corMCAR) on the grand mean (colored line) and confidence interval (shaded area) with respect to the results of fully informed weighted meta‐analyses. Rows show results for the 14 methods to treat missing values (c.f. Table 1). Columns show result for the log response ratio, Hedges' *d* and Fisher's *z* effect sizes with 10% (top) up to 90% (bottom) of standard deviations (*SD*s) and/or sample sizes (SSs) removed. Each panel shows the deviation of the grand mean and its approximated 95% confidence interval (divided by two for better visibility) from the results obtained with a fully informed weighted meta‐analysis. Deviations to the right indicate lower values and deviations to the right indicate higher values

In Figures 3, 4, 5, 6, we show the results of treating increasing proportions of missing *SD*s and/or *SSs* on the grand mean of the three investigated effect sizes (log response ratio, Hedges' *d,* and Fisher's *z*). The different Figures 3, 4, 5, 6 correspond to the four deletion/correlation scenarios (MCAR, MAR, MNAR, and corMCAR) and are similarly organized in the style of a row‐by‐column matrix. The 14 rows correspond to the 14 options to treat missing data (labeled on the right and described in Table 1). The seven columns correspond to the three effect sizes (log response ratio, Hedges' *d,* or Fisher's *z*) and the type of data deleted (only *SD*s, only SSs or both). Fisher's *z* was weighted by SSs alone and thus only those could be deleted. Within each figure, every cell corresponds to a specific combination of the effect size investigated, the type of data deleted and the treatment applied. Within each cell, we show how an increasing proportion of deleted data (from 10% at the top to 90% at the bottom) leads to deviations of the grand mean (solid colored line) and one side (i.e., 50%) of its corresponding confidence interval (dotted lines) from the estimates of a fully informed weighted meta‐analysis (solid black line). Colored lines that match the solid black line indicate that the respective treatment of missing data leads to grand means and confidence intervals that strongly resemble those from fully informed weighted meta‐analyses.

### 3.3 Exploration of simulation results

A summary of the findings regarding the effects of different options to handle missing *SD*s and/or SS in meta‐analysis data sets are listed in Table 2. As a general observation, the deviation introduced by the omission of studies with missing *SD*s and/or *SSs* (with regard to fully informed weighted analyses) mostly exceeded the deviation from all other options to treat those missing data. Unweighted analysis yielded grand means and confidence intervals similar to fully informed weighted analyses except for the case of a correlation between effect sizes and effect size precision. The same holds for the sample‐size weighted analysis. Imputing missing data introduced the least deviation in the log response ratio data set, followed by the correlation coefficient data set and the strongest deviation in the Hedges' *d* data set. Missing *SD*s introduced larger deviations than missing SSs with regard to fully informed weighted analyses. Imputing data missing not at random (MNAR) in the Hedges' *d* data set lead to deviations that are similar to those from the omission of studies with missing *SD*s and/or *SSs*.

**TABLE 2:** Summary of the observed effects of the outlined 14 options to treat missing standard deviations (*SD*s) and/or sample sizes (SSs) on the estimated grand means and confidence intervals in comparison to the results from fully informed weighted meta‐analyses in four simulated data sets with different patterns of missingness and correlation structures (MCAR – **m**issing **c**ompletely **a**t **r**andom, MAR – **m**issing **a**t **r**andom, MNAR – **m**issing **n**ot **a**t **r**andom and corMCAR – *SD*s/SSs are **cor**related to effect sizes and **m**issing **c**ompletely **a**t **r**andom)

Data set	Option	Effects on the grand mean	Effects on the width of the confidence interval
Figure 3 – MCAR	(1) Complete‐case analysis	Increased in volatility with percentage of missing data	Increased nonlinearly with the percentage of missing data
	(2) Unweighted analysis	Unbiased	Unbiased, except smaller CI for Fisher's *z*
	(3) SS‐weighted analysis	Unbiased	Unbiased
	(4–14) Imputations in general	Unbiased for log response ratio, unbiased and slightly volatile for Hedges' *d* and Fisher's *z*	Unbiased, except for high percentages of missing data
	Random sample imputation	–	Unbiased, except smaller for Hedges' *d*
	Bayes predictive mean matching	–	Increases nonlinearly with the percentage of missing data
Figure 4 – MAR	(1) Complete‐case analysis	Deviation increased nonlinearly with the percentage of missing data	Increased nonlinearly with the percentage of missing data
	(2) Unweighted analysis	Unbiased	Unbiased, except smaller CI for Fisher's *z*
	(3) SS‐weighted analysis	Unbiased	Unbiased
	(4–14) Imputations in general	Unbiased for log response ratio, unbiased and slightly volatile for Hedges' *d* and Fisher's *z*	Unbiased, except for high percentages of missing data
	Random sample imputation	–	Unbiased, except smaller for Hedges' *d*
	Bayes predictive mean matching	–	Increases nonlinearly with the percentage of missing data

Data set	Option	Effects on Grand Mean (GM)	Effects on approximated Confidence Interval (CI)
Figure 5 – MNAR	(1) Complete‐case analysis	Increased in volatility with the percentage of missing data. Deviation increased nonlinearly with the percentage of missing *SD*s for Hedges' *d*	Increased nonlinearly with the percentage of missing data
	(2) Unweighted analysis	Unbiased	Unbiased, except smaller CI for Fisher's *z*
	(3) SS‐weighted analysis	Unbiased	Unbiased
	(4–14) Imputations in general	Unbiased for log response ratio. Slightly volatile for Fisher's *z*. Deviation increased nonlinearly with the percentage of missing *SD*s for Hedges' *d*	Unbiased for log response ratio. Decreased with the percentage of missing data for Fisher's *z*. Increased nonlinearly from a threshold of 50%–60% of missing data for Hedges' *d*
	Random sample imputation	–	Unbiased, except smaller for Hedges' *d*
	Bayes predictive mean matching	–	Increased nonlinearly with the percentage of missing data
Figure 6 – corMCAR	(1) Complete‐case analysis	Increased in volatility with the percentage of missing data	Increased nonlinearly with the percentage of missing data
	(2) Unweighted analysis	With deviation	Unbiased, except smaller CI for Fisher's *z*
	(3) SS‐weighted analysis	With deviation	Unbiased
	Mean value, median value, and random sample imputation	Deviation increases approximately linearly with the percentage of missing data, most strongly for missing *SD*s and for Fisher's *z*	Unbiased, except smaller CI for Hedges' *d*
	Linear regression	Unbiased below ca. 80% of missing data	Unbiased, except larger above ca. 70% of missing both, *SD*s and SSs
	Predictive mean matching, classification and regression trees, random forest imputation	Unbiased below ca. 40%–60% of missing data, then with increasing deviation and volatile with the percentage of missing data	Unbiased, except larger above ca. 70% of missing both, *SD*s and SSs
	Bayes predictive mean matching	Unbiased below ca. 50% of missing *SD*s in the log response ratio, ca. 80% of missing *SD*S in Hedges' *d* and 80% in Fisher's *z*	Unbiased, except larger above ca. 70% of missing both, *SD*s and SSs
	Bootstrap expectation maximization	Unbiased until a threshold of ca. 50% of missing data, above which the algorithm frequently failed to converge	Unbiased
	Nonparametric random forest	Unbiased below ca. 70%–80% of missing *SD*s in the log response ratio, nearly linear increase in deviation with the percentage of missing *SD*s in Hedges' *d* and missing SSs in Fisher's *z*	Unbiased
	Additive regression and bootstrap predictive mean matching	Unbiased until a threshold of ca. 50%–70% of missing *SD*s in the log response ratio and in Hedges' *d*, above which the algorithm frequently failed to converge. Unbiased below ca. 60%–70% of missing data in Fisher's *z*	Unbiased

Compared to all other imputation methods, mean, median, and random sample imputation yielded the largest deviation in grand mean estimate and Bayes predictive mean matching yielded the largest increase in the confidence interval. Imputation via bootstrap expectation maximization and additive regression and bootstrap predictive mean matching frequently failed above a threshold of ca. 60% of missing data.

## 4 DISCUSSION

Missing variance measures are a prevalent problem in research synthesis (Gurevitch et al., 2018). Yet, few ecological meta‐analyses have adapted imputation algorithms to handle missing values (Figure 1). Our study demonstrates how the omission of incompletely reported studies (complete‐case analysis), generally increases the confidence intervals and how it results in deviating (potentially even biased) grand mean estimates if *SD*s/SSs are not missing completely at random. The R‐code used to simulate and compare the effects of different meta‐analysis data sets structures, patterns of missingness, and options to handle missing data is freely available at github.com/StephanKambach/SimulateMissingDataInMeta‐Analyses. Although our number of ten replicates is at the lower end of the desired replications in simulation studies (Morris et al., 2019), it was enough to show the general effects of treating missing *SD*s and SSs and meta‐analysis data sets.

In accordance with previous publications (Morrissey, 2016; Nakagawa & Lagisz, 2016), we found that unweighted analyses yielded grand mean estimates that were unbiased with regard to fully informed weighted analyses as long as effect sizes and their corresponding variance estimates were normally and independently distributed. The same holds for sample‐size‐approximated effect sizes variances. In case of a potential relationship between effect sizes and effect size precision (maybe due to different study designs), we advise to apply imputation methods to fill missing *SD*s and/or SSs.

If *SD*s and/or SSs are both MCAR and unrelated to effect sizes, the imputation of up to 90% of missing data yielded grand means similar to those obtained from fully informed weighted meta‐analyses. Below a threshold of ca. 50%–60% of missing *SD*s and/or SSs, imputation methods performed equally or outperformed complete‐case, unweighted, and sample‐size weighted analyses. Yet, our results also demonstrated that different imputation methods can accommodate different data set structures regarding missingness and correlation patterns. Mean, median, and random sample imputations are easy to implement but biased in case of a relationship between effect sizes and effect size precision. Methods applying predictive mean matching tend to suit such relationships but tend to yield a larger confidence intervals of the grand mean. Thus, for any meta‐analysis, the method used to deal with missing *SD*s and/or SSs should be chosen under the following considerations.

### 4.1 The effect size measure

The calculation of the small‐sample bias‐corrected log response ratio and Hedges' *d* both rely on the *SD* values of the control and treatment group. Imputing missing *SD*s thus affects both, effect sizes and effect size weights. For the simple log response ratio and Fisher's *z,* the imputation of missing *SD*s and/or SSs only affects effect size weights.

### 4.2 The type of missing data

Our simulations show that missing SSs could/should routinely be imputed, albeit with caution in case a correlation between effect sizes and sample sizes in the Fisher's *z* data set. Some studies might not report their actual SSs but rather give some indication on the lower or upper boundary (e.g., if an unknown number of samples were excluded from the presented analyses). Such information can be used to curtail the range of imputed values, as can be done within the following imputation methods: Linear regression, predictive mean matching, classification and regression trees, random forest, Bayes predictive mean matching and bootstrap expectation maximization.

For the log response ratio and Hedges' *d,* the treatment of missing *SD*s will have a stronger effect on the grand mean and its confidence interval than the treatment of missing SSs. What we did not investigate with our simulations is the effect of the range and distribution of *SD*s and/or SSs. Larger ranges and nonuniform distributions of *SD*s and/or SSs might likely result in higher variability of imputed values and thus larger confidence intervals. Meta‐analyses that summarize findings from different study designs; for example, across observational and experimental studies or across different organism groups; could harbor exceeding and uneven distributions of *SD*s and/or SSs that we did not simulate in for this study.

### 4.3 The mechanism leading to the observed pattern of missingness

Following our simulation results, data that are missing completely at random (MCAR) or missing at random (MAR) could/should routinely be imputed. For Hedges' *d*, data that are not missing at random (MNAR) introduced deviation in the grand mean (in comparison with a fully informed weighted meta‐analysis), regardless of the option to treat such missing data. Imputation via bootstrap expectation maximization might yield a weaker deviation in grand means, but the applied algorithm frequently failed if more than 60% of *SD*s and/or SSs were missing. Manually fine‐tuning of the respective algorithm parameters might increase its succession rate.

### 4.4 Relationships between effect sizes and *SD*s

Imputation methods that applied a predictive model, that is, except of mean, median, and random sample value imputations, could account for a relationship between effect sizes and effect sizes precision. In case of such a relationship, those algorithms that used predictive mean matching tended to yield grand means that were most similar to the results from fully informed weighted analyses. In case of correlated effect sizes and SSs in the Fisher's *z* data set, the imputation of missing data via mean, median, random sample, and nonparametric random forest imputation introduced a stronger deviation of the grand mean than the omission of those incompletely reported studies.

### 4.5 Summary

Multiple imputation of missing variance measures can be expected to become a standard feature to increase the quality and trustworthiness of future meta‐analyses, as advocated by Gurevitch et al. (2018) and Nakagawa et al. (2017) Our results clearly show that complete‐case and unweighted analyses, although frequently applied, can potentially lead to deviation in the grand means and thus biased conclusions and should therefore be replaced with or (at least) compared to the results of multiple imputation analyses. The same imputation methods might also be applied re‐evaluate the robustness of already published meta‐analyses.

With our simulation study, we aim to raise more awareness on the problem of incompletely reported study results (Gerstner et al., 2017; Parker,Nakagawa, et al., 2016) and their frequent omission in ecological meta‐analyses. Our results discourage the use of complete‐case, unweighted, and sample‐size weighted meta‐analyses since all three options could result in deviation of the grand means and confidence intervals. Even in the absence of valid predictors for the imputation of missing *SD*s or SSs, their imputation has the advantage of including all incompletely reported effect sizes while at the same time preserving the weights of the reported ones.

In summary, our study provides compelling evidence that future meta‐analyses would benefit from a routine application of imputation algorithms to fill unreported *SD*s and SSs in order to increase both, the amount of synthesized effect sizes and the validity of the derived grand mean estimates. The provided R‐script number three could thereby be used to quickly assess to what degree the results of one's own meta‐analysis might be affected by the different options to treat missing *SD*s and SSs.

## CONFLICT OF INTEREST

The authors declare to have no conflict of interest.

## AUTHOR CONTRIBUTIONS

**Stephan Kambach:** Conceptualization (equal); data curation (equal); formal analysis (equal); investigation (equal); methodology (equal); software (equal); supervision (equal); visualization (equal); writing – original draft (equal). **Helge Bruelheide:** Conceptualization (equal); funding acquisition (equal); methodology (equal); supervision (equal); writing – review and editing (equal). **Katharina Gerstner:** Conceptualization (equal); methodology (equal); writing – review and editing (equal). **Jessica Gurevitch:** Conceptualization (equal); methodology (equal); writing – review and editing (equal). **Michael Beckmann:** Conceptualization (equal); methodology (equal); writing – review and editing (equal). **Ralf Seppelt:** Conceptualization (equal); funding acquisition (equal); methodology (equal); supervision (equal); writing – review and editing (equal).

## Supporting information

Data S1

Appendix S1

Appendix S2

## Data Availability

Data sharing not applicable—no new data generated.
