# Supplementary material for: Consequences of multiple imputation of missing standard deviations and sample sizes in meta‐analysis
Source: Ecol Evol. 2020 Oct 7;10(20):11699–712. doi: 10.1002/ece3.6806 (PMC7593147; doi:10.1002/ece3.6806)
Supplement: Supplementary file 2 — Appendix S1 [file ECE3-10-11699-s002.docx]

# Search Queries:

**Web of Science Search:**

- Conducted on: 23.03.2018, 11:30
- Search term: TOPIC: ((meta-analys* OR meta-regression*) AND ecolog*)
- Filters: Timespan: All years. Indexes: SCI-EXPANDED, SSCI, A&HCI, CPCI-S, CPCI-SSH, BKCI-S, BKCI-SSH, ESCI, CCR-EXPANDED, IC.
- Results: 1,744 entries

**Google Scholar Search:**

- Conducted on: 23.03.2018, 11:40
- Sear term: (meta-analys* OR meta-regression*) AND ecolog*
- Results: we extracted the first 980 entries

# These searches yielded 2.626 entries that were screened for the following

# Inclusion criteria:

# the research field was ecology (excluding medical, social, financial and ecosystem service studies),

# the authors conducted an original meta-analysis that was based on summary statistics from previous publications (excluding theoretical, methodological, commentary, raw data analysis and update studies),

# quantified effect sizes as either response ratios, mean differences or correlation coefficients and

# could or should have applied a weighting scheme to summarize those effect sizes (excluding, for instance, meta-analyses that summarized responses of different species that cannot be weighted)

# Data extracted

- ID
- Publication Date
- Type of effect sizes
  - response ratios
  - mean differences
  - correlation coefficients
- Did they encounter missing variance or sample size information in primary studies
  - Yes (variance or sample size?)
  - Stated that they included only completely reported studies
  - No
  - NA
- If data is missing, what percentage is missing in?
  - Primary studies
  - Effect sizes
  - NA
- How did they handle missing variance or sample size?
  - Complete-case analysis
  - Imputation
  - Unweighted analysis
  - Multiple imputation of missing values
  - NA


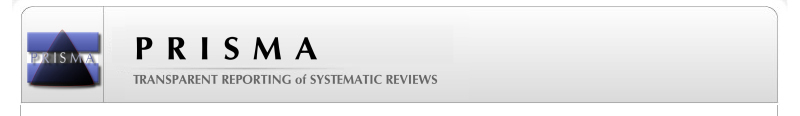
**PRISMA 2009 Flow Diagram**

Additional records identified through other sources
(n = 0)

Records identified through database searching
(n = 2,626)

## Identification

## Screening

## Eligibility

Records and full-text articles excluded with reasons
(n = 2,053)

Records after duplicates and inaccessible were removed
(n = 2,558)

## Included

Studies included in the literature survey
(n = 505)

*From:*  Moher D, Liberati A, Tetzlaff J, Altman DG, The PRISMA Group (2009). *P*referred *R*eporting *I*tems for *S*ystematic Reviews and *M*eta-*A*nalyses: The PRISMA Statement. PLoS Med 6(7): e1000097. doi:10.1371/journal.pmed1000097

**For more information, visit** [**www.prisma-statement.org**](http://www.consort-statement.org/)**.**
