# Supplementary material for: Consequences of multiple imputation of missing standard deviations and sample sizes in meta‐analysis
Source: Ecol Evol. 2020 Oct 7;10(20):11699–712. doi: 10.1002/ece3.6806 (PMC7593147; doi:10.1002/ece3.6806)

S3: Supplementary equations used to calculate the small-sample bias corrected log response ratio (Lajeuness 2015) and Hedges’ *d* for group comparisons and Fisher’s z for correlations together with the corresponding variance estimates. *x_t_* and *x_c_* denote the mean, *sd_t_* and *sd_c_* denote the standard deviation and *n_t_* and *n_c_* denote the sample size in the treatment and control groups whereas *r* and *n* denote the correlation coefficient and sample size.

eqn 1 $\log\mathrm{RR}=\log\left( \frac{x_{t}}{x_{c}} \right)+\frac{1}{2}(\frac{\left( {sd}_{t} \right)^{2}}{n_{t}x_{t}^{2}}-\frac{\left( {sd}_{c} \right)^{2}}{n_{c}x_{c}^{2}})$

eqn 2 $Hedges' d=\frac{x_{t}-x_{c}}{\sqrt{\frac{\left( n_{t}-1 \right){sd}_{t}^{2}+\left( n_{c}-1 \right){sd}_{c}^{2}}{n_{t}+n_{c}-2}}}*J, where J=1-\frac{3}{4\left( n_{t}+n_{c}-2 \right)-1})$

eqn 3 $\mathrm{Fishe}r^{'}s z=0.5*log(\frac{1+r}{1-r})$

eqn 4 ${Var}_{logRR}=\left( \frac{{sd}_{t}^{2}}{n_{t}x_{t}^{2}}+ \frac{{sd}_{c}^{2}}{n_{c}x_{c}^{2}} \right)+\frac{1}{2}(\frac{\left( {sd}_{t} \right)^{4}}{n_{t}^{2}\bar{x}_{t}^{4}}+\frac{\left( {sd}_{c} \right)^{4}}{n_{c}^{2}\bar{x}_{c}^{4}})$

eqn 5 ${Var}_{Hedges' d}=\frac{n_{t}-n_{c}}{n_{t}x_{t}^{2}}+ \frac{{Hedges^{'}d}^{2}}{2(n_{t}+n_{c}})$

eqn 6 ${Var}_{Fisher^{'}s}=\frac{1}{n-3}$

Lajeunesse, M.J. (2015) Bias and correction for the log response ratio in ecological meta-analysis. *Ecology*, **96**, 2056–2063.

Forest plots showing the distribution of effect sizes in the simulated datasets for the four deletion/correlation scenarios: missing completely at random (MCAR), missing at random (MAR), missing not at random (MNAR) are shown in the upper panel. The data sets with correlations between effect sizes and SDs/SSs are shown in the lower panel.


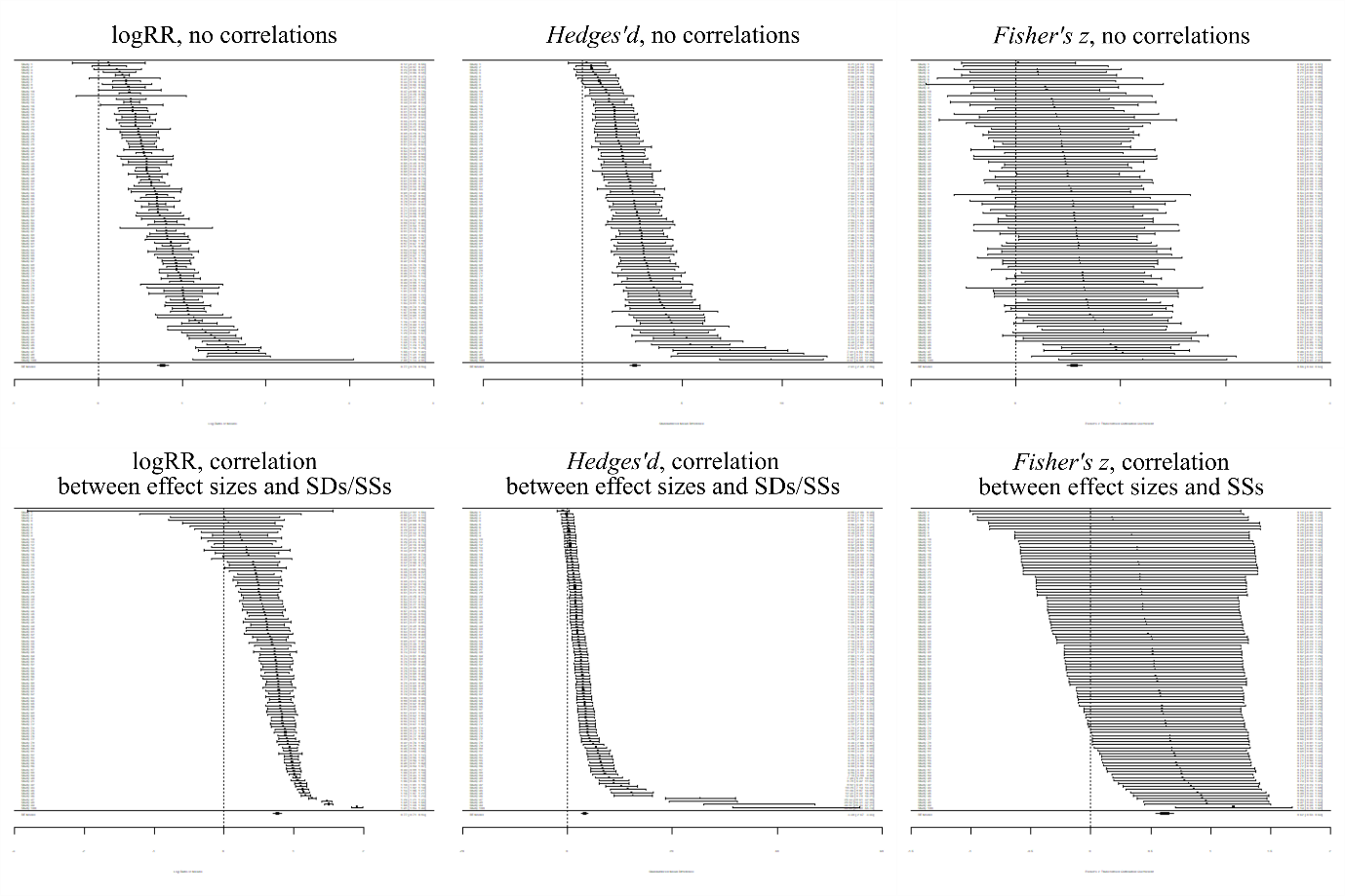

Supplement: Supplementary file 3 — Appendix S2 [file ECE3-10-11699-s003.docx]
